# Telemedicine-based outpatient consultations for hypertension management in rural areas of Kazakhstan

**DOI:** 10.1186/s12913-026-14603-1

**Published:** 2026-04-23

**Authors:** Ainur Bilmakhanbetova, Rebecca Wurtz, Gulnara Kulkayeva, Telman Seisembekov, Serik Ibraev, Aigerim Sipenova, Galiya Orazova, Assiya Turgambayeva

**Affiliations:** 1 NJSC “Astana Medical University”, Beibitshilik St 49/A, Astana, 010000 Kazakhstan; 2 University of Minnesota School of Public Health, Minneapolis, MN USA; 3 Salidat Kairbekova National Research Centre for Health, Astana, Kazakhstan; 4 National Research Oncology Center, Astana, Kazakhstan

**Keywords:** Telemedicine, Hypertension, Rural healthcare, Digital health, Telecardiology, Healthcare access

## Abstract

**Background:**

Hypertension is a leading cause of cardiovascular morbidity and premature mortality worldwide. Limited access to specialist care in rural areas contributes to suboptimal blood pressure control and persistent healthcare disparities. Telemedicine offers a potential solution to bridge this gap.

**Methods:**

This retrospective observational study assessed the feasibility of WhatsApp-based teleconsultations for managing arterial hypertension in rural Kazakhstan. The intervention was conducted in three remote villages served by a district medical center. Patients with uncontrolled hypertension or cardiovascular symptoms were referred for remote cardiology consultations via WhatsApp, integrated with the national electronic health record system. Data on clinical decisions, treatment modifications, and patient self-monitoring behaviors were analyzed. A patient satisfaction survey was conducted between September and December 2024.

**Results:**

A total of 78 patients with arterial hypertension were included. Most consultations were conducted at patients’ homes using smartphones. Stage 3 hypertension was observed in 63% of participants. Treatment adjustments were made in 73.1% of cases following teleconsultation. Gender differences were identified in self-monitoring practices, with women more likely to perform regular home blood pressure monitoring. Among 54 respondents, 82% reported convenience of teleconsultations, and 63% expressed high satisfaction with the quality of care.

**Conclusions:**

Mobile-based teleconsultation using widely available messaging platforms is a feasible and acceptable approach that may improve access to specialist care. This model demonstrates potential for integration into primary healthcare systems to support hypertension management in resource-limited environments. Further studies are needed to evaluate clinical outcomes and long-term effectiveness.

**Trial registration:**

Not applicable.

## Introduction

Hypertension, which affects one in three adults worldwide, is a life-threatening condition that can lead to strokes, heart attacks, heart failure, and kidney damage [1]. In Kazakhstan, cardiovascular diseases remain a leading cause of mortality; in 2022, the mortality rate from diseases of the circulatory system was 154.4 per 100,000 population, including 50.5 deaths per 100,000 from stroke [2].

These data highlight the need for improved access to specialist care, particularly for patients living in rural and underserved areas. In Kazakhstan, arterial hypertension represents a significant public health burden, while blood pressure control remains suboptimal, particularly in rural populations with limited access to specialist care [3].

Access to specialized medical care is more limited in rural areas than in urban areas of Kazakhstan, which leads to inequality in receiving nationally guaranteed free and advanced medical care. A key problem affecting the whole country is the lack of qualified specialists, which forces rural residents to seek consultation in regional referral centers located at a considerable distance, often hundreds of kilometers away.

In addition, rural health care faces constraints including insufficient wireless Internet access, remoteness from pharmaceutical centers, and insufficient medical technology and the expertise to operate and maintain equipment.

To address these challenges, the government of Kazakhstan launched the “Modernisation of Rural Health Care” program in 2022 [4]. This initiative aligns with the WHO-endorsed people-centered primary healthcare model, which emphasizes accessibility, continuity of care, and patient-centered services [5]. As part of this strategy, telemedicine has been introduced to improve access to specialist care for rural populations [6].

Despite infrastructure limitations, mobile phone use is widespread in Kazakhstan, including rural areas [7], and messaging applications such as WhatsApp are commonly used [8, 9]. This makes mobile-based telemedicine a practical and accessible option for delivering specialist consultations in underserved regions.

This study aimed to evaluate the feasibility of WhatsApp-based cardiology teleconsultations for the management of arterial hypertension in rural areas of Kazakhstan. While WhatsApp has been explored for healthcare delivery in other settings, its use in structured telemedicine services for rural hypertensive patients in Kazakhstan has been limited.

## Materials and methods

This was a retrospective observational study designed to evaluate the feasibility of WhatsApp-based cardiology teleconsultations for the management of arterial hypertension in rural Kazakhstan.

**Fig. 1:**
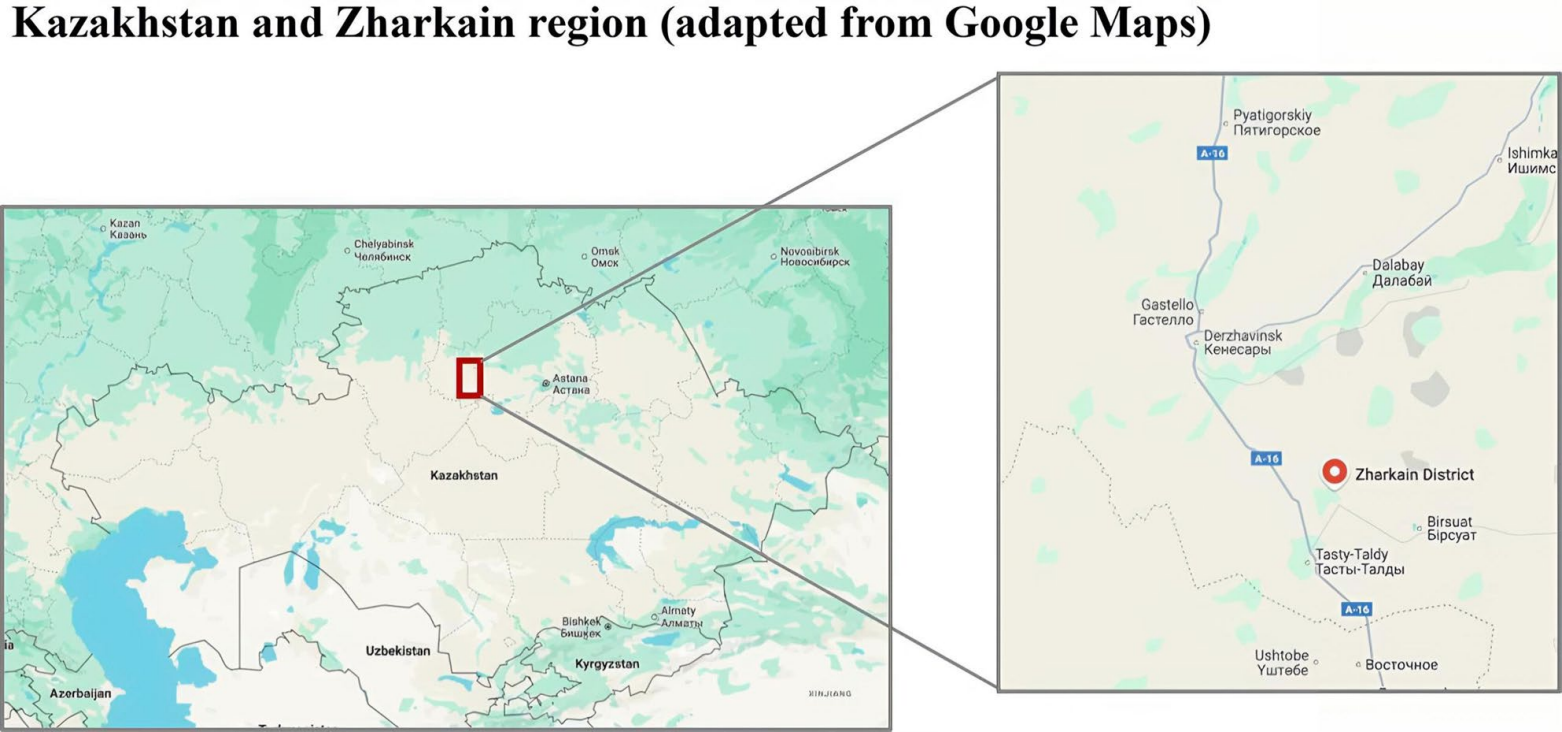
Map of Kazakhstan and the Zharkain district (zoomed-in view) used in the telemedicine project

The telemedicine consultation project was conducted from July 2023 to January 2024. The project included patients from three villages — Taldy-Tasty, Otradnaya, and Pyatigorsk — in the Zharka district, with a total population of 1,326 people, located approximately 500 km from the capital, Astana. This population is served by the district medical center in Derzhavinsk. The geographic coverage of the study is illustrated in Fig. 1.

Patients with arterial hypertension were identified, registered, and referred for teleconsultation by a local general practitioner (GP) based on clinical necessity. A total of 78 patients were included in the study.

This study was conducted in compliance with national telemedicine regulations (Order No. ҚР ДСМ-12, 2021) [6].

The teleconsultation component represented routine clinical practice conducted in accordance with national regulations.

The patient satisfaction survey component was reviewed and approved by the Ethics Committee of the Salidat Kairbekova National Scientific Center for Health Development (Protocol No. 1, dated 6 September 2024).

All participants who completed the survey provided informed consent prior to participation. The study was conducted in accordance with the principles of the Declaration of Helsinki.

The cardiologist (A.B.) developed the telemedicine cardiology program and conducted site visits to Derzhavinsk to introduce the initiative and collaborate with the local GP. During these visits, some of the participating patients were examined in person.

Inclusion criteria included patients with arterial hypertension requiring cardiology consultation due to uncontrolled blood pressure, cardiovascular symptoms, or the need for treatment optimization or preoperative assessment. Exclusion criteria included patients requiring emergency or immediate inpatient care, who were referred for in-person evaluation. Only clinically stable patients were included in the study.

Clinical data, including electrocardiography (ECG), lipid profile, glucose, creatinine levels, and urinalysis, were recorded in the national integrated health information system (NHIS), Damumed (CIT Damu LLC, Astana, Kazakhstan), and used to support clinical decision-making. The GP issued teleconsultation requests through the system.

Each teleconsultation lasted approximately 30–35 min. Prior to the consultation, the cardiologist reviewed the patient’s electronic medical record, including medical history, laboratory results, and ECG data.

During the teleconsultation, a comprehensive clinical assessment was performed, including evaluation of symptoms and blood pressure control. Clinical decisions were based on patient-reported symptoms, medical history, and available diagnostic data.

Management decisions included optimization of antihypertensive therapy, recommendations for further diagnostic testing when necessary, and follow-up planning. Patients were referred for in-person evaluation if clinically indicated.

Feasibility outcomes included completion of teleconsultations, successful connection, and the need for assistance. Clinical variables included blood pressure control, patient-reported symptoms, and changes in antihypertensive therapy.

The cardiologist, located in Astana, was notified of scheduled consultations via the NHIS and reviewed patient data prior to each session. The GP informed patients of consultation times. WhatsApp was used solely as a communication tool for video consultations. No medical data were stored within the application. All consultations followed standard clinical and confidentiality principles.

For four patients without smartphones, family members assisted by providing access to a mobile device.

### Statistical analysis

Statistical analysis was performed to describe patient characteristics and to explore differences between male and female participants. Variables included demographic characteristics (age, sex), clinical features, comorbidities, self-management practices, and teleconsultation outcomes.

Categorical variables were expressed as frequencies and percentages, and continuous variables as means ± standard deviations. Comparative analyses between female and male participants were conducted using appropriate statistical tests. Specifically, chi-square or Fisher’s exact tests were applied for categorical variables, depending on expected cell counts, and the independent samples t-test was used for continuous variables.

Given the descriptive nature of the study and the relatively small sample size, analyses were limited to unadjusted comparisons. Not all variables were subjected to inferential testing; p-values are reported only for selected variables where statistical comparison was considered appropriate.

A p-value < 0.05 was considered statistically significant.

Data were managed and analyzed using Microsoft Excel.

## Results

### Patient characteristics

A total of 78 patients with arterial hypertension were included in the study. Baseline demographic and clinical characteristics are presented in Table 1. Most consultations (76/78) were conducted in the patient’s home setting. The majority of patients (63%) had stage 3 hypertension. Comorbid conditions included diabetes mellitus and chronic kidney disease, both of which were more prevalent among male participants.

**Table 1:** Characteristics of patients and teleconsultation outcomes (*n* = 78)

Characteristics	Female (*n* = 54)	Male (*n* = 24)	*p*-value
Sex, n (%)	54 (69%)	24 (31%)	—
Age, mean (SD)	59 (1.7)	63 (2.2)	0.298
Place of consultation, n (% within gender)			
Home	45 (83%)	13 (54%)	—
Workplace	9 (17%)	9 (38%)	—
Hospital	0	2 (8%)	—
Diagnosis, n (% within gender)			
Evaluation at request of GP; no hypertension confirmed	1 (2%)	3 (13%)	—
AH Stage 1	1 (2%)	1 (4%)	—
AH Stage 2	16 (29%)	4 (17%)	—
AH Stage 3	35 (65%)	14 (59%)	—
Hypertension associated with pregnancy	1 (2%)	0	—
Hypertension associated with chronic pyelonephritis	0	2 (8%)	—
Medical conditions, n (% within gender)			
Diabetes mellitus	3 (6%)	4 (17%)	0.113
Ischemic heart disease	18 (33%)	12 (50%)	0.163
Chronic kidney disease	1 (2%)	2 (8%)	0.169
Atrial fibrillation	5 (9%)	3 (13%)	0.663
Medical self-management, n (% within gender)			
Electronic blood pressure monitor at home	50 (93%)	17 (71%)	0.011*
Self-monitoring of blood pressure	48 (89%)	17 (71%)	0.048*
Taking antihypertensive medication	46 (85%)	20 (83%)	0.834
ECG available in NHIS	50 (93%)	24 (100%)	0.171
Teleconsultation results, n (% within gender)			
Cleared for scheduled surgery	3 (6%)	3 (13%)	—
Hypertension treatment modification	45 (83%)	12 (49%)	—
Recommended for further GP evaluation	1 (2%)	1 (4%)	—
— Inpatient treatment	0	4 (13%)	—
— Coronary angiography	5 (9%)	3 (13%)	—
— Coronary artery bypass grafting after angiography	0	1 (4%)	—

Abbreviations: SD – standard deviation; AH – arterial hypertension; NHIS – national health information system

* *p* < 0.05 considered statistically significant

A total of 74 patients (94.9%) owned smartphones, while four used devices borrowed from family members. WhatsApp was available on all devices, and all video consultations were initiated by the cardiologist.

### Feasibility and implementation outcomes

Teleconsultations were successfully conducted using WhatsApp in all cases, demonstrating the feasibility of this approach in a rural setting. Most patients were able to participate from home, and real-time interaction between patient and cardiologist was consistently achieved.

A significantly higher proportion of women (50; 90%) owned an electronic blood pressure monitor compared to men (17; 71%) (*p* = 0.011). Among those with a home device, 74% of women reported regular self-monitoring, compared to 26% of men. During consultations, patients measured their blood pressure and shared readings via the smartphone camera.

Most patients (85%) were receiving antihypertensive therapy; however, 15% reported occasional non-adherence.

### Clinical observations during teleconsultations

Treatment regimens were adjusted in 57 patients (73.1%) based on clinical assessment during teleconsultations, reflecting a frequent need for therapy optimization identified in routine practice.

Medication-related side effects included persistent dry cough associated with ACE inhibitors (enalapril) and peripheral edema associated with calcium channel blockers (amlodipine). These symptoms resolved following treatment adjustments.

Patients were advised to perform structured self-monitoring of blood pressure, and treatment decisions were individualized based on reported measurements. In some cases of resistant hypertension, substantial modification of therapy was required.

One case of pregnancy-associated hypertension resolved after delivery.

Inpatient evaluation was recommended for 13 patients due to severe hypertension or suspected ischemic heart disease (e.g., chest pain, reduced exercise tolerance, dyspnea). All were referred for further diagnostic evaluation, including coronary angiography. One patient was diagnosed with critical (95%) left main coronary artery stenosis and underwent urgent coronary artery bypass grafting (CABG).

These findings represent clinical observations within a feasibility study design and should not be interpreted as evidence of clinical effectiveness.

### Patient-reported acceptability

Among survey respondents, 82% reported that teleconsultations were convenient, 63% were fully satisfied with the quality of care, and 75% appreciated the ability to receive care from home without travel or waiting times. These findings support the acceptability of telemedicine in rural populations.

## Discussion

This study demonstrates the feasibility and practical implementation of a telecardiology model designed to improve access to specialist care for patients with arterial hypertension in rural settings. The model was based on structured patient selection through primary care physicians, with all patients registered in national electronic health systems (NHIS/Damumed), ensuring continuity of care and integration into routine clinical workflows.

The use of WhatsApp as a communication tool proved to be practical and accessible, particularly in a population with limited digital resources. Its widespread availability and familiarity, including among older adults, eliminated the need for additional infrastructure, training, or specialized equipment. This highlights the feasibility of implementing low-cost telemedicine solutions to address inequalities in access to specialist care and provide timely cardiology support in rural populations [10].

The high prevalence of stage 3 hypertension observed in this study may reflect delayed referral to specialist care and long-term management within primary care settings without cardiology consultation. Many patients had a history of markedly elevated blood pressure levels (≥ 180 mmHg) that had not been formally reclassified prior to specialist evaluation. Following teleconsultation, reassessment of clinical status and home blood pressure measurements led to reclassification of hypertension severity and optimization of treatment according to current guidelines [3, 9].

Similarly, the high rate of treatment adjustments (73.1%) likely reflects gaps in baseline management, including limited access to cardiology expertise, reliance on monotherapy, and challenges in regular follow-up in rural settings. This finding is consistent with global evidence demonstrating suboptimal hypertension control, particularly in low- and middle-income countries. Large-scale studies such as the PURE study have shown that a substantial proportion of patients with hypertension remain untreated or inadequately controlled, especially in resource-limited settings [11]. Similarly, previous research has highlighted significant gaps in hypertension awareness, treatment, and control in LMICs, as well as disparities in access to care and specialist services [12, 13]. These findings support the role of telemedicine as a practical tool to bridge these gaps and improve access to guideline-based care.

Importantly, the findings suggest that limited access to specialist care in rural settings may contribute to delayed recognition of disease severity and suboptimal management of hypertension. Teleconsultation provided an opportunity for reassessment of clinical status, correction of treatment strategies, and timely referral when necessary, thereby strengthening the link between primary and specialized care.

Gender-related differences observed in this study may reflect both demographic and behavioral factors. Women were more frequently represented among older patients, which may be associated with higher life expectancy. In contrast, men appeared less engaged in follow-up care and less likely to participate in consultations. These differences may be explained by variations in health-seeking behavior, as men are often less likely to seek timely medical attention and may demonstrate lower adherence to long-term management of chronic conditions. Similar gender disparities in hypertension control and self-care behaviors have been described in previous studies [14].

The implementation of telecardiology consultations enabled timely outpatient interventions, including adjustment of antihypertensive therapy and referral for inpatient evaluation when indicated. This approach helped to overcome geographical barriers and improve access to specialist care in remote regions. Notably, in one case, teleconsultation contributed to the detection of critical left main coronary artery stenosis, leading to timely surgical intervention.

This is supported by recent evidence demonstrating that remote patient management remains effective regardless of rurality and travel distance. For example, a sub-analysis of the TIM-HF2 trial showed that telemedicine-based care maintained its effectiveness even among patients living in remote areas, highlighting its potential to overcome geographic barriers to care [15].

The findings of this study are consistent with growing evidence from low- and middle-income countries, where widely available messaging platforms such as WhatsApp are increasingly used to support healthcare delivery. Their low cost, accessibility, and ease of use make them particularly suitable for rural and resource-limited settings, supporting patient follow-up, remote consultations, and chronic disease management [10, 14, 16].

The findings of the patient satisfaction survey indicate a high level of acceptability of teleconsultations among rural patients. The majority of respondents reported convenience and satisfaction with the quality of care, highlighting the potential of mobile-based telemedicine to overcome geographical barriers and improve access to specialist services [10].

Overall, this model demonstrates that a simple and scalable telemedicine approach may improve access to cardiology care and may support guideline-based management of hypertension in underserved rural populations.

Importantly, these findings should be interpreted within the context of a feasibility study design and do not provide evidence of clinical effectiveness.

## Limitations

This study has several limitations. First, it was conducted without a control group, which limits the ability to draw conclusions about clinical effectiveness. The study primarily reflects a feasibility and organizational model rather than an outcome-based evaluation. Second, follow-up duration was limited, and quantitative data on blood pressure dynamics after treatment adjustments were not systematically collected.

Third, the sample size was relatively small and restricted to selected rural settlements, which may affect the generalizability of the findings. Fourth, patient satisfaction data were self-reported and collected using a non-validated questionnaire, which may introduce response bias. Fifth, variability in digital literacy and access to technology may have influenced participation, particularly among older individuals. Finally, patient selection was based on primary care physician referral, which may introduce selection bias. The gender imbalance in the sample (with a higher proportion of female participants) should also be considered when interpreting the findings.

Despite these limitations, the study provides valuable real-world insights into the feasibility and implementation of telemedicine in rural healthcare settings.

### Implications for future research

Future research should include controlled study designs with larger and more diverse populations to evaluate the clinical effectiveness of telecardiology interventions. Longitudinal studies are needed to assess the impact of remote consultations on blood pressure control and cardiovascular outcomes.

In addition, future work should explore strategies to improve patient engagement, particularly among men, and assess the integration of telemedicine into national healthcare systems. From a policy perspective, scaling such low-cost digital solutions may help reduce disparities in access to specialist care in rural and resource-limited settings.

## Conclusion

This study demonstrates the feasibility and practical applicability of a telecardiology model for the management of arterial hypertension in rural areas of Kazakhstan. The use of teleconsultation enabled timely access to specialist care, reduced the need for long-distance travel, and supported clinical management of chronic cardiovascular. High patient acceptability further supports the use of this approach in resource-limited rural populations.

These findings highlight the potential of simple, widely available mobile-based solutions to improve access to care and reduce healthcare disparities. The results provide real-world evidence supporting the integration of telemedicine into routine clinical practice in settings with limited specialist availability.

Further research with controlled study designs is needed to evaluate clinical effectiveness, long-term outcomes, and cost-effectiveness. Future efforts should focus on scaling teleconsultation services, integrating them into national healthcare systems, and expanding such models to other regions facing similar healthcare access challenges.

## Data Availability

The datasets used and analyzed during the current study are available from the corresponding author on reasonable request.

## Abbreviations

BP: Blood pressure
GP: General practitioner
EHR: Electronic health record
AH: Arterial hypertension
NHIS: National Health Information System
ECG: Electrocardiogram
